# ChatGPT in dermatology: exploring the limited utility amidst the tech hype

**DOI:** 10.3389/fmed.2023.1308229

**Published:** 2024-01-11

**Authors:** Zhuoya Zhang, Jiale Zhang, Lianyuan Duan, Cheng Tan

**Affiliations:** ^1^Affiliated Hospital of Nanjing University of Chinese Medicine, Nanjing, China; ^2^Institute of Basic Theory for Chinese Medicine, China Academy of Chinese Medical Sciences, Beijing, China; ^3^Nanjing Hongtu Artificial Intelligence Technology Research Institute, Nanjing, China

**Keywords:** dermatology, dermatology education, artificial intelligence (AI), ChatGPT, GPT-4, medical education

## Introduction

The emergence of ChatGPT has ignited debates within the medical community regarding its potential to supplant human doctors (1). From a dermatological perspective, it may be premature to indulge in such discourse. Before immersing ourselves in the question of whether ChatGPT can “supplant” human dermatologists, it is crucial to ascertain if ChatGPT is a genuinely useful tool in dermatology or merely an overhyped technology. This will determine if busy dermatologists should devote their time to learning and mastering its use to enhance their clinical or research capabilities.

## Methods

To delve into ChatGPT's role in dermatology, we conducted a comprehensive literature search across three databases: PubMed, Web of Science, and Embase. Specifically, a basic keyword search in PubMed was conducted using (ChatGPT) AND (dermatology). In Web of Science, the search criteria were ChatGPT [All Fields] AND dermatology [All Fields]. Similarly, for Embase, we employed the terms “ChatGPT”/exp AND “dermatology”/exp. ZYZ and JLZ independently screened the initial results, first reviewing titles and abstracts, followed by a meticulous full-text review to refine the selection. In disagreements, CT's expertise was sought to reach a consensus. Emphasis was placed on the quality and relevance of the articles. Qualitative studies were assessed against the COREQ checklist (2), ensuring transparency. Pilot studies adhered to the CONSORT Extension for Pilot and Feasibility Trials (3). Non-epidemiological articles were chosen based on their novelty, recency, and non-redundancy. Our objective was to spotlight fresh perspectives on ChatGPT's potential in dermatology. This systematic approach yielded distinct articles, which we subsequently categorized as positive, neutral, or skeptical. A detailed breakdown of these articles is provided in Table 1.

**Table 1:** Perspectives on ChatGPT in dermatology: a compilation from 14 selected studies.

**Title**	**Authors (top 3)**	**Perspectives**
**Positive applications**		
Artificial intelligence-derived dermatology case reports are indistinguishable from those written by humans: a single-blinded observer study (4)	Dunn, C., J. Hunter, W. Steffes	AI-derived dermatology case reports match human-written ones.
ChatGPT- Quo Vadis? (5)	Kaliyadan, F., and K. A. Seetharam	Applications include image diagnosis (clinical and histological), secretarial support, social media improvements for practitioners, research, and medical education.
An original study of ChatGPT-3.5 and ChatGPT-4 Dermatological Knowledge Level based on the Dermatology Specialty Certificate Examinations (6)	Lewandowski, M., P. Łukowicz, D. Swietlik	ChatGPT shows high dermatological knowledge, with GPT-4 outperforming GPT-3.5.
Trends in accuracy and appropriateness of alopecia areata information obtained from a popular online large language model, ChatGPT (7)	O'Hagan, R., R. H. Kim	Improving trend in newer iterations indicates potential for both patients and dermatologists.
Performance of ChatGPT on dermatology Specialty Certificate Examination multiple choice questions (8)	Passby, L., N. Jenko, and A. Wernham	GPT-4 achieved a 90% score on Dermatology exams.
Artificial Intelligence in Medical Education: Comparative Analysis of ChatGPT, Bing, and Medical Students in Germany (9)	Roos, J., A. Kasapovic, T. Jansen	GPT-4 and Bing surpassed student performances in.
**Neutral perspectives**		
Dermatology in the wake of an AI revolution: who gets a say? (10)	Beltrami, E. J., and J. M. Grant-Kels	ChatGPT shows potential in educational tools, but limitations exist in generating board-style dermatology questions.
ChatGPT for healthcare providers and patients: Practical implications within dermatology (11)	Jin, J. Q., and A. S. Dobry	ChatGPT may streamline clinical tasks, boost patient and medical education, support clinical research, and raise healthcare literacy, but its accuracy and overview of available evidence require rigorous human supervision.
Comparing Meta-Analyses with ChatGPT in the Evaluation of the Effectiveness and Tolerance of Systemic Therapies in Moderate-to-Severe Plaque Psoriasis (12)	Lam Hoai, X. L., and T. Simonart	Potential in data processing for MAs/NMAs, but requires rigorous human oversight due to its limitations in presenting accurate overview.
Using ChatGPT for Writing Articles for Patients' Education for Dermatological Diseases: A Pilot Study (13)	Mondal, H., S. Mondal, and I. Podder	Generates readable patient education text, but text similarity/plagiarism concerns exist.
Genital and Extragenital Lichen Sclerosus et Atrophicus: A Case Series Written Using ChatGPT (14)	P, P. J., S. S. Prasad, and N. Manohar	Fluent communication observed, yet struggled in sourcing reliable literature.
**Skeptical views**		
The complex ethics of applying ChatGPT and language model artificial intelligence in dermatology (15)	Ferreira, A. L., and J. B. Lipoff	AI-generated diagnosis discussions should include bias, consent, privacy, security, and accountability.
Comment on “Performance of ChatGPT on dermatology Specialty Certificate Examination multiple choice questions” (16)	Kleebayoon, A., and V. Wiwanitkit	Concerns include reliance on unreliable data, lack of validation without human review, and potential misuse in exam.
ChatGPT underperforms in triaging appropriate use of Mohs surgery for cutaneous neoplasms (17)	O'Hern, K., E. Yang, and N. Y. Vidal	ChatGPT's performance is subpar for triaging Mohs surgery for cutaneous neoplasm.

## Main findings

Upon examining the varied perspectives on ChatGPT's role in dermatology, it is evident that there is a range of positive and cautious opinions. Several factors contribute to these differences. Firstly, the emerging nature of the technology is a significant factor. As ChatGPT is introduced into the medical field, it is met with enthusiasm and skepticism. Some researchers recognize the potential benefits of such tools in enhancing patient education and aiding in preliminary diagnostics. However, others express concerns about its accuracy and the potential risks of relying on AI-driven systems. Secondly, the research methodologies and data sources used in different studies can influence the outcomes and interpretations. For instance, studies that utilize comprehensive and diverse datasets might report higher efficacy of ChatGPT, while those using more specific or limited datasets might point out its constraints. Additionally, the objectives and context of each study can shape the conclusions drawn. Researchers focusing on the potential of technological advancements in dermatology might have a favorable view of ChatGPT. In contrast, those emphasizing patient safety and data integrity might be more critical. In conclusion, the varied perspectives on ChatGPT's application in dermatology highlight the complexities of integrating new technologies into medical practice. A comprehensive understanding requires further research and interdisciplinary discussions.

**Figure 1:**
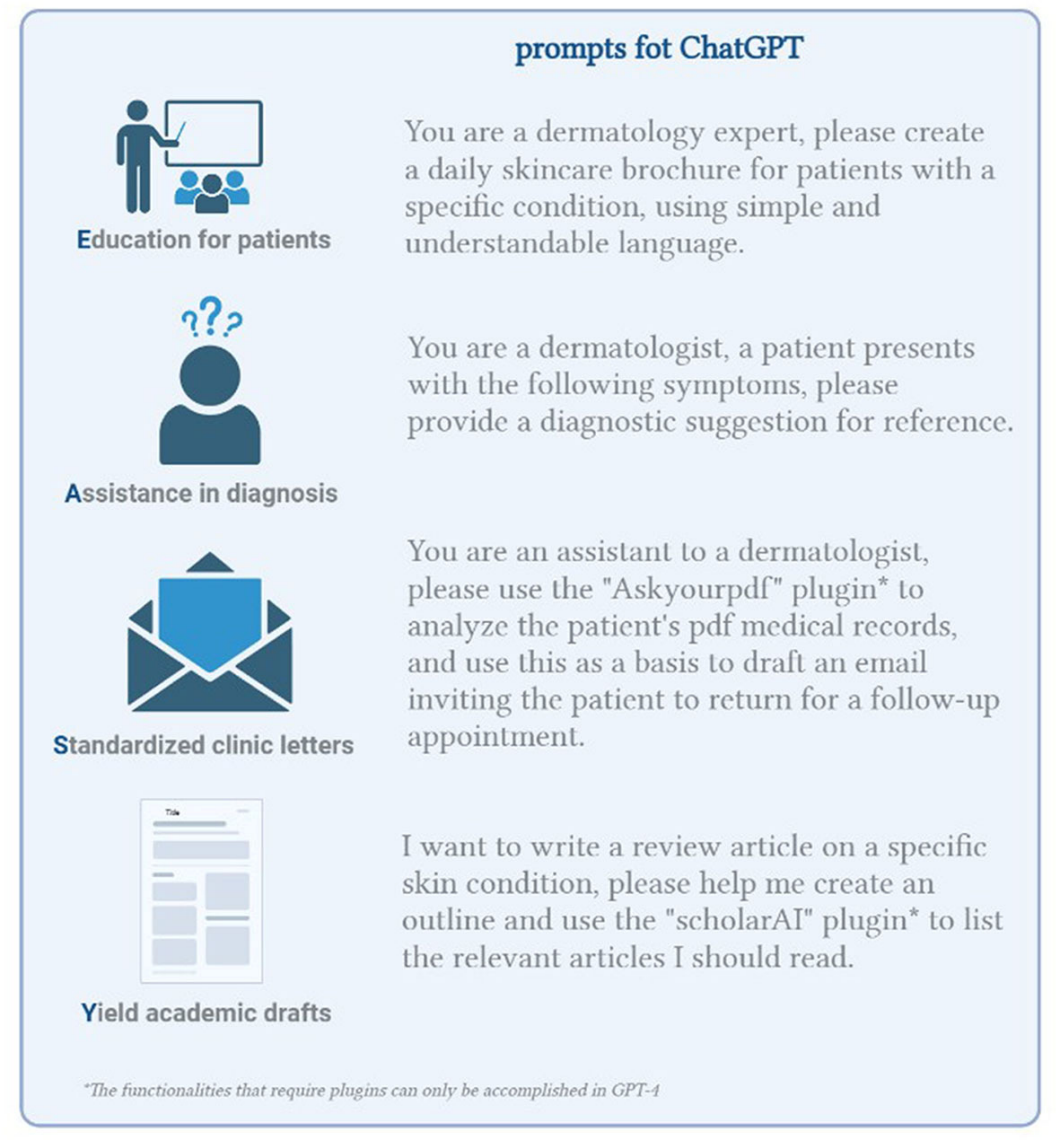
The “Easy” mode and reference prompts for ChatGPT application in dermatology.

Currently, although ChatGPT shows promise, it is unable to deliver the full spectrum of performance it claims. Based on practical experience, we categorize its dermatologist support as “Easy” mode. To illustrate how this mode can be implemented in practice, Figure 1 will present a listing of selected prompts.

### Education for patients

ChatGPT can function as a patient education tool, generating information about various skin diseases or specific aspects. This can manifest as informational leaflets or responses to patients' specific queries about their conditions.

### Assistance in diagnosis

ChatGPT can analyze the data and provide a probable diagnosis by inputting symptoms. However, it is vital to remember that the accuracy of the diagnosis hinges on the quality of the data provided and the AI tool's comprehension of the condition.

### Standardized clinic letters

ChatGPT can generate “standardized” or “custom-made” clinic letters, such as letters informing the patient of a diagnosis or a planned procedure. This can aid in streamlining communication between doctors and patients.

### Yield academic drafts

ChatGPT can provide robust first drafts of academic articles, theses, or assignments. These drafts can then be corrected and refined by the user (18).

In dermatological education, GPT indeed shines with its unique capabilities. First and foremost, GPT's capacity to create interactive dermatological learning tools is noteworthy. Although it may not yet accurately simulate skin lesions, it can generate queries closely resembling those from real dermatological patients. This capability promotes active learning among dermatology practitioners, aiding in developing critical thinking and sharp diagnostic skills.

Moreover, GPT plays a vital role in fostering educational equity in dermatology. GPT's academic plugins can offer substantial support in regions with limited learning resources. In less developed areas, where dermatologists and structured dermatological education are scarce, querying GPT can provide access to comprehensive professional knowledge, potentially breaking the cycle of educational deficits in these regions. Additionally, GPT's strong multilingual proficiency reduces language barriers, promoting accessibility and fairness in knowledge dissemination.

Lastly, GPT has the potential to bring creative perspectives to dermatological education. Unlike human-centric educational approaches that may not cater to a vast and diverse audience, GPT can process and analyze large volumes of information. This capacity enables GPT to uncover patterns and insights that might elude human educators, potentially leading to novel and surprising revelations. This aspect is continuously enhanced with GPT's frequent updates, each iteration improving its understanding of human needs.

## Discussion

### Navigating challenges and ethical landscapes in dermatological AI

ChatGPT provides new possibilities in dermatology and dermatology education, but its contributions are incremental rather than revolutionary, making its role more supplementary than primary.

#### Uncertainty and credibility

**Figure 2:**
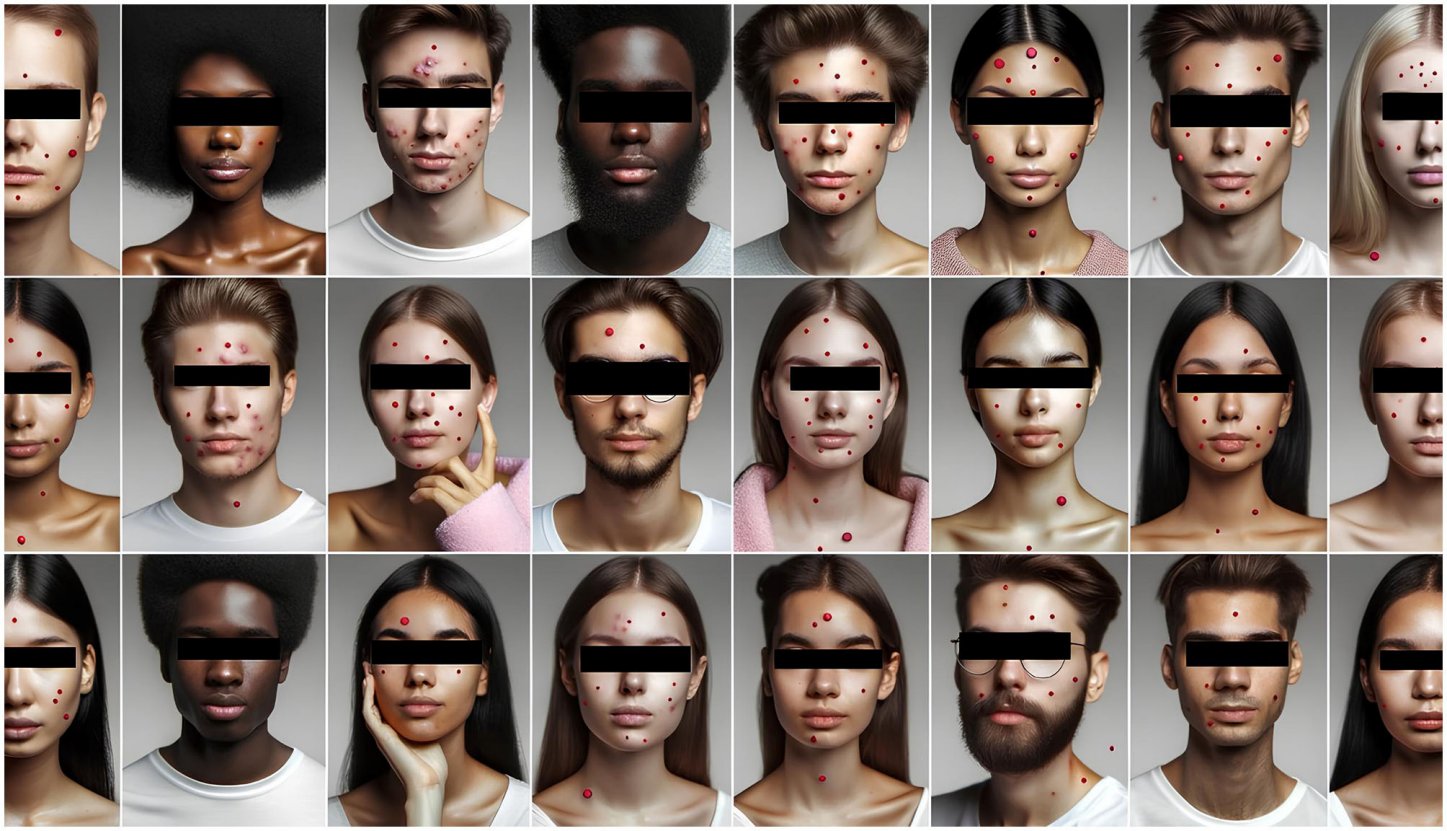
Photo of a diverse group of individuals showcasing various types of acne, with marked spots indicating the acne lesions for educational purposes, created by DALL·E3; the faces in these images are AI-generated, not real people, and eyes are obscured to avoid controversy.

AI models, such as ChatGPT, face significant credibility challenges due to their non-clinical origins and the opaque nature of their generative processes. Their constraints, notably the inability to process visual data, reliance on static information, and uneven linguistic capabilities, further limit their utility in dermatology education. Furthermore, while auxiliary tools like “scholarAI” offer potential benefits in accessing scientific literature, they too come with their own set of limitations. The accuracy and relevance of the content retrieved are not always guaranteed. This risks generating potentially misleading or outdated references, further exacerbating the credibility issue. A poignant illustration of these limitations is seen with the DALL·E3 model. Designed for image generation, DALL·E3 can craft images that are artistically captivating. However, when employed to generate images of acne lesions for pedagogical purposes, the visually striking results often miss the mark in terms of clinical precision and educational value, as shown in Figure 2.

The juxtaposition of these models and tools underscores the imperative need for rigorous clinical validation before their integration into medical practice. Despite the promise they hold, the potential pitfalls cannot be overlooked.

#### Ethical and legal considerations

Integrating AI into dermatology presents intricate ethical and legal challenges. These range from ensuring patient confidentiality to navigating potential legal implications of AI-influenced clinical decisions. Achieving genuine informed consent and maintaining decision transparency are also paramount. An extended dimension of ethical dilemmas emerges from ChatGPT's unique generative capability.

#### Data quality and diversity

GPT models' effectiveness depends on diverse and quality training data. A biased dataset could make AI outputs inadequate or even harmful. To circumvent these pitfalls, an international collaborative approach, involving partnerships with global institutions—including those from rural and typically underrepresented regions—can be monumental in garnering a comprehensive dermatological dataset.

### Strategic solutions for enhancing AI's effectiveness in dermatology

#### Enhancing explainability

Elevating GPT model explainability is crucial to address credibility challenges. This can be achieved by developing interpretable AI frameworks, making algorithmic decisions more transparent and clinically scrutinizable. Besides developing interpretable AI frameworks, it is also imperative to build a user-friendly interface where dermatologists can interactively probe and question the AI's decisions. Establishing visual aids or decision trees that trace back the logic behind an AI's recommendation could be an indispensable tool for clinicians, promoting trust and enhancing the therapeutic decision-making process.

#### Robust ethical and legal frameworks

Establishing robust governance structures is vital for the ethical deployment of AI in dermatology. This should involve safeguarding patient privacy, establishing guidelines for informed consent, and ensuring practitioner accountability.

#### Expanding data acquisition and diversity

Efforts should prioritize expanding dermatological data acquisition and diversifying its representational scope. Collaborative efforts across healthcare entities and promoting advanced data augmentation techniques are essential. While it's essential to have diverse datasets, the quality and veracity of the data cannot be compromised. AI models should be continually validated against new datasets to ensure their robustness and reliability. Additionally, partnerships with global dermatological organizations can facilitate data collection across various ethnicities, enhancing the AI's diagnostic precision across diverse patient populations.

#### Comprehensive training

Dermatologists require rigorous training on the potentials and limitations of AI. This will foster a deeper understanding and ethical considerations of AI deployment. It's not just about understanding the potentials and limitations.

#### Patient-centric engagement

In the AI-augmented healthcare realm, the doctor-patient bond is vital. Upholding informed consent in this AI era goes beyond mere transparency; it's about ensuring patients remain central to their care. The AI tool, dermatologist, and patient should collaboratively work for optimal outcomes. The human touch in dermatology is irreplaceable. While tools like ChatGPT offer diagnostic promise, the essence of patient care—be it the tactile sensation of a skin examination or addressing concerns like acne or vitiligo—demands more than algorithms. These conditions, seemingly trivial to some, deeply impact patients' psychological wellbeing. Addressing them requires a blend of clinical expertise, empathy, and genuine connection.

In our exploration of ChatGPT's role in dermatology, it emerges as a multifaceted tool with its primary strength in the realm of education. Beyond educational purposes, ChatGPT also offers modest benefits in streamlining workflow and administrative tasks within dermatological practice. These aspects, while not as prominent as its educational applications, contribute to overall efficiency enhancements in the field.

The integration of ChatGPT into dermatological education provides interactive and accessible learning experiences, especially valuable in regions with limited educational resources. Its capacity to process and present complex dermatological knowledge in various languages makes it a vital tool in global dermatological education. However, this is juxtaposed with its limitations in accurately processing visual dermatological data, a critical aspect in fields like dermatopathology.

While the allure of ChatGPT is apparent in both educational and administrative contexts, its role should be seen as supplementary. The core of dermatological practice — nuanced expertise and human interaction — cannot be replaced by AI. ChatGPT's current capabilities, while promising, call for a measured integration into dermatological practice and education.

Maintaining the original discussion of limitations, it's clear that ChatGPT, despite its potential, has not yet evolved to become an indispensable tool in dermatology. Its efficacy is constrained by inherent limitations and ethical considerations. As such, its adoption in dermatology should be approached with a blend of enthusiasm and caution, ensuring it complements rather than supplants the essential human elements of medical care.

In conclusion, ChatGPT augments the dermatologist's toolkit in education, workflow, and administrative tasks, but the journey ahead in fully realizing its potential requires a collaborative, informed, and ethically grounded approach. As AI technology continues to advance, it's crucial to continuously evaluate and adapt its integration into dermatological practice and education, ensuring it aligns with the evolving needs and standards of the field.

## Ethics statement

Written informed consent was obtained from the individual(s) for the publication of any identifiable images or data included in this article.

## Author contributions

ZZ: Conceptualization, Writing – original draft, Writing – review & editing. JZ: Conceptualization, Writing – original draft, Writing – review & editing. CT: Conceptualization, Formal analysis, Writing – review & editing. LD: Writing – original draft & review & editing, Conceptualization, Investigation.
